# BatchPrimer3: A high throughput web application for PCR and sequencing primer design

**DOI:** 10.1186/1471-2105-9-253

**Published:** 2008-05-29

**Authors:** Frank M You, Naxin Huo, Yong Qiang Gu, Ming-cheng Luo, Yaqin Ma, Dave Hane, Gerard R Lazo, Jan Dvorak, Olin D Anderson

**Affiliations:** 1 Department of Plant Sciences, University of California, CA 95616, USA; 2 Genetic Resources Conservation Program, University of California, CA 95616, USA; 3 Western Regional Research Center, Agricultural Research Service, US Department of Agriculture, 800 Buchanan Street, Albany, CA 94710, USA

## Abstract

**Background:**

Microsatellite (simple sequence repeat – SSR) and single nucleotide polymorphism (SNP) markers are two types of important genetic markers useful in genetic mapping and genotyping. Often, large-scale genomic research projects require high-throughput computer-assisted primer design. Numerous such web-based or standard-alone programs for PCR primer design are available but vary in quality and functionality. In particular, most programs lack batch primer design capability. Such a high-throughput software tool for designing SSR flanking primers and SNP genotyping primers is increasingly demanded.

**Results:**

A new web primer design program, BatchPrimer3, is developed based on Primer3. BatchPrimer3 adopted the Primer3 core program as a major primer design engine to choose the best primer pairs. A new score-based primer picking module is incorporated into BatchPrimer3 and used to pick position-restricted primers. BatchPrimer3 v1.0 implements several types of primer designs including generic primers, SSR primers together with SSR detection, and SNP genotyping primers (including single-base extension primers, allele-specific primers, and tetra-primers for tetra-primer ARMS PCR), as well as DNA sequencing primers. DNA sequences in FASTA format can be batch read into the program. The basic information of input sequences, as a reference of parameter setting of primer design, can be obtained by pre-analysis of sequences. The input sequences can be pre-processed and masked to exclude and/or include specific regions, or set targets for different primer design purposes as in Primer3Web and primer3Plus. A tab-delimited or Excel-formatted primer output also greatly facilitates the subsequent primer-ordering process. Thousands of primers, including wheat conserved intron-flanking primers, wheat genome-specific SNP genotyping primers, and *Brachypodium *SSR flanking primers in several genome projects have been designed using the program and validated in several laboratories.

**Conclusion:**

BatchPrimer3 is a comprehensive web primer design program to develop different types of primers in a high-throughput manner. Additional methods of primer design can be easily integrated into future versions of BatchPrimer3. The program with source code and thousands of PCR and sequencing primers designed for wheat and *Brachypodium *are accessible at .

## Background

Primer design programs are crucial in optimizing the polymerase chain reaction (PCR). A poorly designed primer can result in little or no target product. Numerous web-based or standard-alone programs for PCR primer design are available but vary in quality and functionality [1,2]. Primer3 [3,4] is the most popular non-commercial primer design software because of its capabilities and free accessibility. Primer3 core program, a C language-written command line program, has great flexibility to optimize a number of parameters such as product size, melting temperature (T_m_), GC content, primer length, 3' end stability, self complementarity, primer dimer possibility, position constraints and so forth to get the best primer pairs, and provides the potential to design different types of PCR primers to meet various needs. Due to complexity of Primer3 core program in parameter input, it is difficult to directly use the command line program to design primers. Primer3Web [3] is the first web interface for Primer3 written in Perl. The interface has a powerful, but complex HTML form, including all of possible parameters and options used in the Primer3 core program. Primer3Plus [5] further reorganized and optimized the Primer3Web's user interface in light of parameter categories and primer design tasks. Primer3Web provided two types of primer design, generic primers and hybridization oligos. Primer3Plus further expanded to have cloning and sequencing primer design as well as a primer management module to facilitate further primer analysis and ordering. Several other web-based or command line pipeline programs using Primer3 as a primer design engine also have been developed [6-10]. However, most of those web-based programs lack batch primer design capability. For many large-scale primer design projects, in addition to the requirement of suitable primer design methods, two additional features, batch input of DNA sequences and primer ordering ready output are necessary.

Simple sequence repeat (SSR) and single nucleotide polymorphism (SNP) are two types of important genetic markers. Large numbers of SSRs and SNPs have been detected in various species and used in genetics and breeding [11-15]. A number of different SNP genotyping technologies have been developed based on various methods of allelic discrimination and detection platforms (see review [15]). Primer extension is the most commonly used approach to SNP genotyping because it can be used in a wide variety of high-throughput detection platforms, i.e., electrophoresis, fluorescence resonance energy transfer, fluorescence polarization, arrays, mass spectrometry, and luminescence [15]. A primer extension reaction involves two types of primer design: single base extension primers and allele-specific primers. A software tool for designing SSR flanking primers and SNP genotyping primers in a high-throughput mode is increasingly needed.

On the basis of the Primer3 core program, Primer3Web [3] and Primer3Plus [5], we developed a new web-based application, BatchPrimer3. The aims of BatchPrimer3 development are (1) to implement additional options in primer design, (2) to improve capability of the program to process a large number of DNA sequences, and (3) to provide convenient primer outputs for viewing primer details, printing primer lists, editing primers and finally placing primer orders. We extended Primer3Web and Primer3Plus to have batch processing capability of designing primers, and integrated SSR detection and SSR-flanking primer design to have flexible options for SSR search criteria and to export both SSR detection results and SSR-flanking primer list. In addition, we implemented primer design methods for two basic types of SNP genotyping primers, single base extension (SBE) primers and allele-specific (AS) primers, as well as tetra-primers for tetra-primer amplification refractory mutation system (ARMS) PCR [16]. DNA sequencing primer design is also reimplemented in this program. The BatchPrimer3 program is easily extendible and additional primer design methods may be integrated in the future.

## Implementation

### Web application design

**Figure 1:**
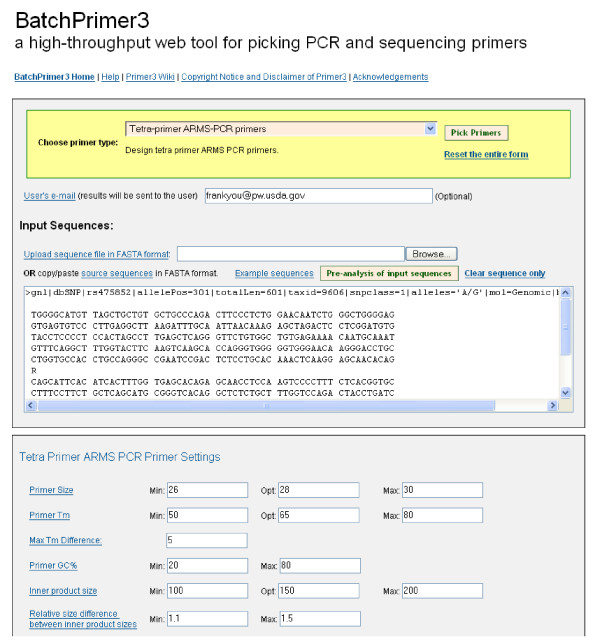
**Web interface of BatchPrimer3 v1.0 application.** Various types of primer design can be selected from the primer type pull-up combo-box, and corresponding parameter setting panels are placed below the sequence input box. Pre-analysis of input sequences can be performed before batch primer design.

BatchPrimer3 was designed as a web application consisting of a set of CGI programs written in Perl, which can run on different operating systems, such as Solaris, Linux, Mac OS or Windows with an Apache HTTP server and Perl interpreter program. The open source program Primer3Web [3] was adopted as a start point. The similar interface in Primer3Plus [5] was used, which has a pull-down combo-box for primer type selection, and a text field together with a button for uploading a sequence file (Figure 1). This task-orientated interface [5] with modular programming design provides extendibility to integrate new primer design methods to the program. File uploading allows users to input a large number of target sequences for batch primer design and overcomes the sequence size limit in an HTML textarea field. The pre-analysis module of input sequences is added to calculate sequence properties, such as sequence lengths and GC contents, which is helpful to determine parameter ranges for primer design. The parameter setting panels are customized according to different primer types. When a user chooses a primer type, the corresponding parameter setting panels are represented directly below the sequence input box (Figure 1). An email address text field is also provided to allow a user to receive an email alert of primer design results.

The Primer3 core program [3] is used to be the major primer design engine for picking the best pairs of standard PCR primers. An additional primer-picking algorithm was implemented to select position-restricted primers such as SBE primers, AS primers and sequencing primers. The best primers are selected based on the quality scores of candidate primers. The quality score is a weighted linear function of primer length, T_m_, GC content, number of a single-base repeat and simple sequence repeats, number of an ambiguity code (N), and self-complementarity of the entire primer and the last 10 nucleotides in the 3' end. The maximum quality score of a candidate primer is 100. If the parameter values of a candidate primer are beyond the user-specified ranges, or a candidate primer contains single sequence repeats, the quality score is set to 0. Within specified parameter range, the closer the user-specified optimum value is to a calculated primer property value, the higher is the primer quality score. If the highest score is zero, no primer is given for the specified criteria. The T_m _value of a primer varies in different T_m _calculation models – this often results in different set of primers being picked even when using the same T_m _parameter settings [17]. In BatchPrimer3, the T_m _of generic primers, hybridization oligo, and SSR flanking primers is calculated using Primer3 core program. An additional T_m _calculation module was implemented for SNP genotyping primers and sequencing primers based on the same model of the nearest neighbour thermodynamic theory [18,19] as used in the Primer3 core program (v 1.1) [4].

### Primer design strategy

Besides generic primer and hybridization oligo [3,5], sequencing primer design is reimplemented in a batch mode. SSR flanking primer design and several SNP genotyping primer designs are newly implemented in BatchPrimer3 v1.0.

#### SSR screening and primer design

SSR is a simple repeat of short motifs, 1 to 6 base pairs in length with at least 12 nucleotides in length of SSR [13]. Options of di- to hexa-nucleotide repeat motifs and minimum repeat numbers for each type of motifs are provided in the web interface. An SSR detection algorithm was adopted from the SSR search program [20] to detect the SSR motifs which are then masked as targets. The Primer3 core program is then used to pick the best pairs of primers that flank the targets. If more than one SSR is detected in the same sequence, separate pairs of SSR primers will be designed for each SSR.

#### Design of primers flanking SNPs

In most SNP detection platforms, SNP detection requires previous PCR amplification of the genomic region that flanks the SNP site. BatchPrimer3 v1.0 provides a module to design pairs of primers that flank the SNP site.

#### Design of primers for SNP genotyping

In BatchPrimer3 v1.0, three types of SNP genotyping primers can be designed: (1) SBE primers, (2) AS primers, and (3) tetra-primers for tetra-primer ARMS PCR system [16].

##### SBE primer design

**Figure 2:**
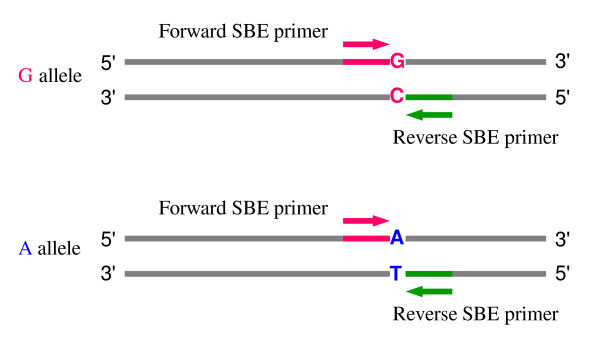
**Primer design of single base extension (SBE) primers.** One SBE primer is positioned at the base of the 3' end immediately upstream to the SNP.

SBE primers are widely used in some high-throughput detection technology platforms, such as SNaPshot (Applied Biosystems) and fluorescence polarization detection (FP-TDI) [21,22]. An SBE primer that anneals immediately adjacent to the SNP is extended by one base using a fluorescently labeled ddNTP (Figure 2). For each SNP, it is possible to design two SBE primers, one for each orientation (forward and reverse). For each orientation, all the primer candidates meeting the user-specified primer length range (greater than or equal to the minimum size and less than or equal to the maximum size) are picked. Then the T_m_, GC content and quality score of each candidate are calculated. The primer with the highest score is chosen. A pair of SNP flanking primers and SBE primer can be designed in the same module.

##### AS primer design

**Figure 3:**
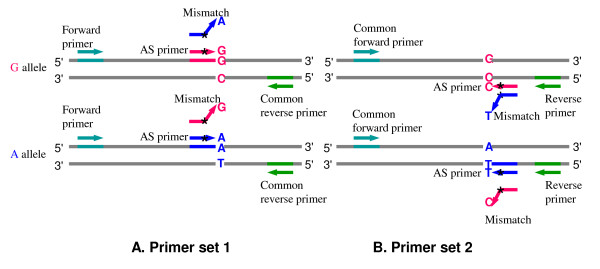
**Primer design of allele-specific (AS) primers.** Two AS primers, one for each allele of a SNP are designed. The AS primers contain one of two polymorphic nucleotides at the primer 3' end. Two sets of primers, either forward or reverse primers can be designed. If a common reverse or forward primer is used in a PCR reaction, the reaction is called allele-specific PCR (AS-PCR). Generally, two PCR reactions are needed for detection of both alleles of a SNP. A variant of AS-PCR is to use only one AS primer and two outer SNP-flanking primers in a single PCR reaction, i.e., three-primer nested system [27]. A mismatch (represented by *) may be deliberately introduced at the third position from the 3' end of each of AS primers to increase allelic specificity (See Table 1). The SNP **R **(**G**/**A**) is illustrated as an example, and the other types of SNPs can be applied in the same way.

SNPs can be genotyped using AS primers with the last nucleotide at the 3' end of a primer corresponding to the site of the SNP [23]. In the AS extension reaction, two primers are required, one for each allele of a SNP (Figure 3). AS extension relies on the difference in extension efficiency of DNA polymerase between primers with matched and mismatched 3' ends. DNA polymerase extends a primer only when the 3' end is perfectly complementary to the DNA template. Thus, an AS primer is specific to one of two alleles of a SNP at the 3' end of primers and specifically amplifies one of the two alleles. Genotyping is based on determination of the primer that produces the amplicon [15]. If a common reverse primer is used in the reaction, the reaction is called allele-specific PCR (AS-PCR) [24-28]. Typically two forward AS primers are used in AS-PCR with a shared reverse non-specific primer. Two PCR reactions are needed to detect both alleles of a SNP [25,26,28]. One variant of AS-PCR is to use only one AS primer and two SNP-flanking primers in one PCR reaction (three-primer nested system) [27]. To enhance the specificity in the AS-PCR reaction, an additional mismatch may be deliberately introduced at the third or other position from the 3' end of each of the AS primers [16,24-26]. Rules for selection of a nucleotide for the mismatch [16,24,29] are summarized in Table 1: "a 'strong' mismatch (G/A or C/T) at the 3'-end of an allele-specific primer will likely need a 'weak' second mismatch (C/A, or G/T) and vice versa, whereas a 'medium' mismatch (A/A, C/C, G/G or T/T) at the 3'-end will likely require a 'medium' second mismatch" [16]. An option is provided in the parameter setting panel for adding an additional mismatch and choosing the position of the second deliberate mismatch (the default is the third position). Two sets of AS primers, in both forward and reverse direction can be designed in BatchPrimer3. The SNP-flanking primer pair also can be designed together with AS primers or separately. The same primer selection algorithm is used to choose the AS primers with the highest scores.

**Table 1:** The mismatches at the 3' end and the second mismatches at the third or other position from the 3' end in an allele-specific primer

IUB/IUPAC Code of a SNP	Alleles of a SNP	Mismatch at the 3' end (forward/reverse)	Mismatch strength at the 3' end	Second mismatch at the third or other position from the 3' end
R	G/A	G/T	Weak	G/A, T/C
		A/C	Weak	A/G, C/T
Y	T/C	G/T	Weak	G/A, T/C
		A/C	Weak	A/G, C/T
S	G/C	G/G	Medium	C/C, A/A, G/G, T/T
		C/C	Medium	C/C, A/A, G/G, T/T
W	A/T	A/A	Medium	C/C, A/A, G/G, T/T
		T/T	Medium	C/C, A/A, G/G, T/T
K	G/T	G/A	Strong	G/T, A/C
		T/C	Strong	T/G, C/A
M	A/C	G/A	Strong	G/T, A/C
		T/C	Strong	T/G, C/A

##### Primer design for tetra-primer ARMS PCR

**Figure 4:**
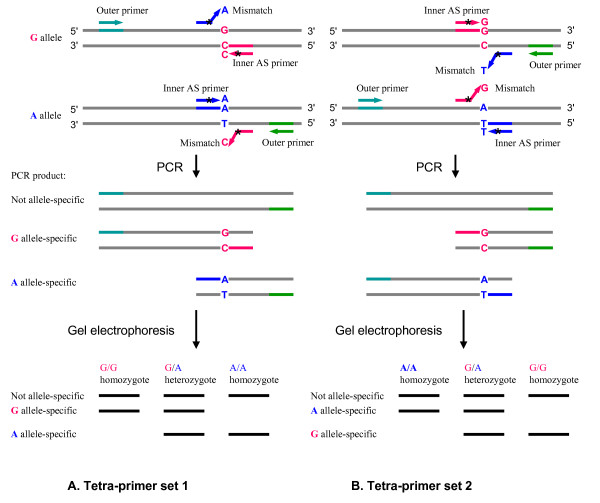
**Schematic illustration of primer design for the tetra-primer ARMS PCR (redraw from Ye et al., 2001 [16]).** The SNP **R **(**G**/**A**) is presented as an example, and the other types of SNPs can be applied in the same way. Four primers, one pair of inner allele-specific (AS) primers and one pair of outer standard primers, are required in a single PCR reaction. Two AS products, one for the **G **allele and the other for the **A **allele are amplified using two pairs of primers. The former consists of a **G **AS primer and an outer standard primer, and other latter contains an **A **AS primer and an outer standard primer. A mismatch (represented by *) is deliberately introduced at the third position from the 3' end of each of the two AS primers to increase allelic specificity (See Table 1). Two outer standard primers are designed in such a way that the amplicons of two alleles differ in sizes and can be resolved by agarose gel electrophoresis.

Ye et al. [16] proposed a simple, effective and economical SNP genotyping method based on AS primers called tetra-primer ARMS-PCR [30-35]. This procedure adopts principles of the tetra-primer PCR method [36] and the amplification refractory mutation system (ARMS) [24]. Four primers are required to amplify a larger fragment from template DNA containing the SNP and two smaller fragments representing each of the two AS products. Primers are designed in such a way that the allelic amplicons differ in size and can be resolved by agarose gel electrophoresis. To enhance the specificity of the reaction, in addition to the first mismatch at the 3' end of AS primers, an extra mismatch is also deliberately introduced at the third position from the 3' end of each of the two inner AS primers (Table 1, Figure 4). From the primer design perspective, two sets of tetra-primers can theoretically be designed for any SNP depending on the AS primer orientation. The schematic diagram of two-set primer design is shown in Figure 4. Although the web program [37] for designing a single set of primers for a SNP is available [16], BatchPrimer3 v1.0 implemented a batch module to easily design two sets of tetra-primers for a SNP.

### Program input

**Figure 5:**
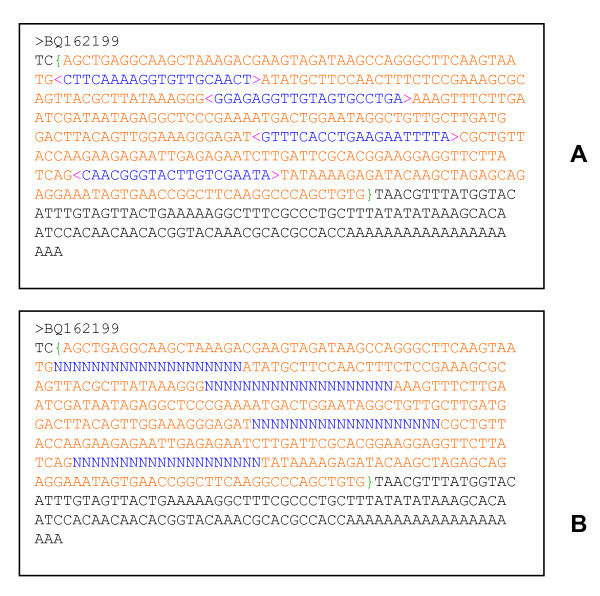
**An example of DNA sequence preprocessing.** Any unwanted regions for primer design in sequences can be masked using a pair of "<>" to keep the sequence unchanged (**A**). Alternatively, the unwanted regions can be replaced with "Ns" (**B**). The included region can be specified by one pair "{}" and only one included region can be masked.

Sequences can be input in two ways. Sequences in FASTA format can be copied and then pasted to the sequence text box (Figure 1). This approach has a maximum size limit of 256 kb. For a large volume of sequences, a FASTA file can be uploaded to the server and the sequence size limitation only depends on Internet speed and server machine memory. When inputting a FASTA file or a single sequence, a header line starting with ">" is mandatory for each sequence. However, empty lines or spaces within sequences are allowed (Figure 1 and 5).

For SNP flanking primer or SNP genotyping primer design, the SNPs or alleles in sequences need to be converted to IUB/IUPAC codes (G/C→S, A/T→W, G/A→R, T/C→Y, G/T→K, A/C→M), and the sequence file follows the NCBI dbSNP FASTA format. If multiple SNPs exist in one sequence, BatchPrimer3 will try to design primers for each. Because only one SNP is taken as target and other SNPs are converted to one of the SNP alleles, we suggest that a separate sequence for each SNP is generated based on a reference sequence.

As in Primer3Web [3] and Primer3Plus [5], for any type of primer design, the "{}" pair can be inserted into sequences to specify an included region (for example, excluding the vector sequence fragments on both ends), and the "< >" pair to specify excluded regions. An example is to mask all introns with "< >" to design primers only in exons (Figure 5A). An alternative method to specify excluded regions is to replace the unwanted regions with "N" (Figure 5B) and set the parameter "Max # Ns" as 0. The " []" pair is adopted to specify targets. If multiple targets are set in one sequence, at least one target will be included in the PCR product [3]. It is notable that target masking can be used only for generic primer design in BatchPrimer3. In addition, multiple targets and excluded regions can be specified in a sequence but only one included region is allowed.

### Program output

**Figure 6:**
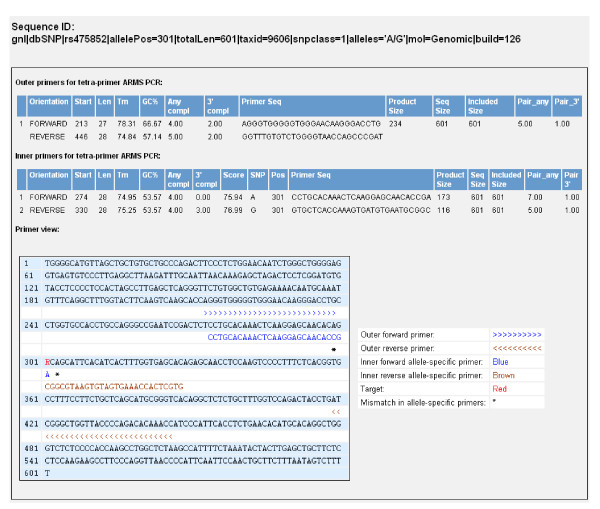
**Screenshot of a primer set in batch primer design.** The picture shows the primer design results of sequence ID (rs16791736) for tetra-primer ARMS PCR.

The BatchPrimer3 program produces four parts of outputs: (1) a main HTML page containing the primer design summary of all input sequences, (2) an HTML table page listing all designed primers and primer properties, (3) a tab-delimited text file with the same contents in the HTML table page, and (4) a detailed primer view page for each sequence with successfully designed primers (Figure 6). A simple click on the links on the main HTML page or HTML table page will display the primer view. The primer list can be directly saved as a text file or an Excel file for further editing or primer ordering. All primer design results can be downloaded as a zipped file.

## Results

Using the BatchPrimer3 program we have designed thousands of primers in several genomic research projects, including conserved intron-flanking primer pairs from EST sequences for wheat SNP discovery, SNP genotyping primers for wheat SNP mapping, primer pairs from *Brachypodium *bacteria artificial chromosome (BAC) end sequences for *Brachypodium *SNP discovery, sequencing primers from EST sequences for gene-specific sequencing, and SSR flanking primer pairs from *Brachypodium *EST and BAC end sequences for *Brachypodium *SSR genotyping. Most of these primers have been validated in experiments from several laboratories.

### Wheat conserved intron-flanking primer design

In the project with a goal to discover SNPs in wheat, deletion-mapped wheat EST contigs [38] were compared with rice genomic sequences using BlastN to detect the splice sites (exon/exon junctions) in ESTs. Intron-flanking primers (called conserved primers) were then designed for PCR amplification and sequencing of introns and the nested portions of exons [39]. A total of 6,045 deletion-mapped contigs and singletons were used to perform BlastN searches. Rice introns were inserted into the ESTs at the predicted positions and replaced with corresponding number of ambiguity codes (Ns) (Figure 5B). PCR primer pairs anchored in neighbouring exons were designed using "Generic primer" module in BatchPrimer3. An additional primer analysis was performed to identify candidate primer pairs that span at least one intron in rice. A total of 2,223 conserved primer pairs were generated. They were further filtered to select only those from single-copy genes. A total of 1,821 of these primer pairs were used for PCR amplification and sequencing of the amplicons from 16 DNAs comprising wheat diploid ancestors and tetraploid wheat in seven different laboratories. Of 240 conserved primer pairs used in one of the laboratories, 228 produced amplicons (95%). All conserved primers were made publicly available and are downloadable from the wheat SNP web site [40].

### Wheat SNP genotyping primer design

Sequences of amplicons produced with the 1,821 conserved primers were used to design genome-specific primers for PCR amplification of target DNA from a single genome of polyploid wheat and sequencing of the amplicons in a panel of wheat lines and synthetic wheats. A total of 1,527 loci containing one or more SNPs were discovered [14]. The SNPs provide a large number of potential SNP markers. Using this population of SNPs, SBE primers, AS primers, and tetra-primers were designed (Table 2). Theoretically two sets of primers for each SNP can be designed according to primer orientation (Figure 2, 3 and 4). Moreover, a gene locus may have several sets of primers for different SNPs or reverse and forward primers. Table 2 lists the number of three types of genotyping primers designed from 1,527 gene loci and their genome and chromosome distribution in wheat. Gene loci rather than sets of primers are reported in Table 2. The primers are derived from 1,186, 1,346, 485 gene loci for the three types of SNP genotyping primers, respectively. The success rates of picking primers based on SNPs was 48.7%, 61.6%, and 12.7% for the three types of primers, respectively, whereas the success rate based on gene loci was 77.7%, 88.2% and 31.8%, respectively. Because the tetra-primers design requires similar primer properties in two outer primers and two inner AS primers and differing sizes in two inner products, fewer tetra-primers were obtained than of the other two primer types. These primers will be a valuable resource for wheat genetics and breeding. All are accessible at [41]. In addition, 450 SBE primers and corresponding SNP-flanking primer pairs were designed for diploid *Aegilops taushii *SNPs for their mapping using the SNaPshot assay (Applied Biosystems) (Luo et al., unpublished data). The default parameters for PCR primer design were used, and SBE primers were designed in a range of 25 to 35 bases in primer length, 50 to 90°C in T_m _and 20 to 80% in GC content. Success rate of SBE primers in PCR amplification was 82.4% (371 out of 450 primer sets).

**Table 2:** The number of wheat SNP genotyping primers designed from 1,527 gene loci containing one or more SNPs.

Primer type	Genome	Chromosome							Total
			
		1	2	3	4	5	6	7	
SBE primer	A	51	57	43	63	53	55	54	376
	B	52	51	47	66	54	53	59	382
	D	58	57	56	56	60	84	57	428
	
	Total	161	165	146	185	167	192	170	1186

AS primer	A	62	69	49	69	63	62	62	436
	B	64	55	54	65	60	65	68	431
	D	67	63	61	67	66	90	64	479
	
	Total	193	187	164	201	189	217	194	1346

Tetra- primer	A	17	19	16	25	22	22	20	141
	B	20	25	14	19	25	26	27	156
	D	23	23	23	27	30	42	20	188
	
	Total	60	67	53	71	77	90	67	485

Note: The same parameters were applied to SBE primer and AS primer design: primer length of 25 to 30 bases with the optimum 27 bases, T_m _of 50 to 65°C with the optimum 60°C. All three types of primers shared the same GC content (20% to 80%), primer complementarity (≤ 8) and 3' complementarity (≤ 3). Tetra primer design required a primer of 28 bases in length, ranging from 26 to 30 bases, and a broader T_m _range (50 to 80°C) with the optimum 65°C. The optimum inner product size was 120 bases ranging from 100 to 200 bases with a proper size ratio range of two products (from 1.1 to1.5).

### *Brachypodium *standard primer design

In the *Brachypodium *SNP discover project, the non-redundant *Brachypodium *(accession Bd21) BAC end sequences [42,43] were used to design standard PCR primers for DNA amplification in the accessions Bd21 and Bd3-1, to find SNPs by comparing sequences of the two lines [44]. A total of 960 pairs of primers were designed, 689 (71.8%) successfully amplified single products. Approximately one quarter (28.2%) of the primers failed to amplify a product in Bd3-1 while producing an amplicon in Bd21.

### *Brachypodium *SSR detection and SSR-flanking primer design

To develop SSR markers, the 49,134 *Brachypodium *BAC end sequences [43] were screened for SSRs and the corresponding SSR-flanking primers were designed. Screening was performed for di-, tri-, tera-, penta- and hexa-nucleotide repeats. The minimum SSR length was set to 12 base pairs and the minimum number of SSR motif repeats were 6, 4, 3, 3, 3, respectively, for di- to hexa-nucleotide repeats. Default parameters for primer design were used: product size of 100 to 300 bases, primer size of 18 to 23 bases with the optimum size of 21 bases, T_m _of 50 to 70°C with the optimum at 55°C and the maximum difference of 20°C, and the primer GC content set at 30 to70%. A total of 10,064 SSRs (1,123 dinucleotide, 3,928 trinucleotide, 3,818 tetranucleotide, 819 pentanucleotide and 376 hexanucleotide) were detected and 8,977 pairs of SSR primers were successfully designed. Genotyping of those SSRs is in progress. The primer list is available at [45].

### Performance of BatchPrimer3 web application

Performance of the BatchPrimer3 program depends on primer type, speed of the server on which BatchPrimer3 resides, client Internet speed (affecting sequence data loading to the server) as well as the efficiency of the BatchPrimer3 and Primer3 core programs. Primer design for generic primer, sequencing primer, and SBE primer performs faster than for other types of primers, with tetra-primer design taking the most time. For example, the above screening of 49,134 sequences for SSR and subsequent primer design took about 526 seconds, whereas the tetra-primer designs from 5,509 sequences took 432 seconds through Internet connections.

## Discussion

### Parameter setting

To obtain high quality primers, primer length, T_m_, GC content, specificity, and intra- or inter-primer homology must be taken into account [2]. Primer specificity is related to primer length and the final 8 to 10 bases of the 3' end sequence. A primer length of 18 to 30 bases is optimum [1,2]. T_m _is closely correlated to primer length, GC content and primer base composition. Ideal primer T_m _is in the range of 50 to 65°C with GC content in the range of 40 to 60% for standard primer pairs [1,2,17]. However, the optimal primer length varies depending on different types of primers. For example, SNP genotyping primers need a longer primer length (25 to 35 bases) to enhance their specificity, and thus the corresponding T_m _might be higher than 65°C. A suitable T_m _can be obtained by setting a broader GC content range (20 to 80%). A broader GC content range can increase the success rate of primer picking from sequences with relatively low GC contents (AT rich species or sequences). In BatchPrimer3, the entire primer complementarity and 3' complementarity between and within primers are calculated to assess the intra- or inter-primer homology for the entire primer or 10 bases at the 3' end. Generally, the score measuring the entire primer complementarity should be less than or equal to 8 and the score for 3' end complementarity should be less than or equal to 3 [3].

### Batch primer design

The advantage of batch primer design is its high efficiency. However, designing primers in the batch mode can result in a failure to design primers for some sequences because input sequences vary in sequence quality and properties (for example, AT rich or GC rich) and/or because the same set of primer design parameters cannot be applied to all sequences. A utility tool for pre-analysis of input sequences is therefore provided in BatchPrimer3 to help users to understand the basic properties of input sequences, such as sequence length and GC content in an entire set of input sequences. The information can be used to adjust the parameter ranges of product size and primer GC content.

The success rate of picking primers in the batch primer design mode is affected by sequence quality, target polymorphism location (for SNP genotyping primers), and parameter settings. Ambiguity codes (N) in a sequence may result in a failure in picking proper primers, especially for position-specific primer design. SBE and AS primers are picked from the region adjacent to, or including the target polymorphism. If an ambiguity code exists in the region or the T_m_, GC content or other primer properties cannot meet the parameter settings, primer design will fail. In tetra-primer ARMS PCR, two inner AS products are amplified, which requires that the polymorphism site cannot be too close to an end of a sequence. For SSR primer design, no proper primer is available if an SSR is located at the end of a sequence.

The source of input sequences affects PCR amplification. EST sequences are often used to design SSR primers [11] or other types of primers (such as sequencing primers and conserved primers, see **Results**). A low amplification rate was reported for EST-derived SSR primers [11] and one of the possible reasons is that one or both primers of the EST-derived SSRs traverse a splice site [11]. The splice site analysis of EST sequences should be performed to mask splice sites or to insert ambiguity codes (Ns) into EST sequences at a splice site (Figure 5B). The wheat conserved primer design strategy is a successful example to resolve this problem.

## Conclusion

BatchPrimer3 is a comprehensive, extendible web primer design program to design different types of PCR and sequencing primers. The batch sequence input and convenient tab-delimited primer outputs facilitate rapid primer design for a large number of sequences and primer ordering. Additional primer design methods can be easily integrated into the program in the future. Using this software program, thousands of primers for wheat and *Brachypodium *SNP discovery, and SNP and SSR genotyping, have been designed and validated. The program with source code and designed primers can be accessed at [41] (also see Additional file 1).

## Availability and requirements

**Project name: BatchPrimer3**.

**Project home page: **.

**Operating systems: **the software should run in different operating systems, such as Solaris, Linux, Mac-OS or Windows. Tests were performed in Solaris and SuSE Linux systems.

**Programming language: **Perl

**Other requirements: **Apache HTTP server, Perl interpreter program

**License: **GNU PGL

**Any restrictions to use by non-academics: **None

## Authors' contributions

FMY developed the major modules of the BatchPrimer3 program, designed wheat conserved primers and SNP genotyping primers, and drafted the manuscript. NH and YQG validated wheat conserved primers and *Brachypodium *primers. M–CL and YM designed and evaluated wheat SBE primers. DH implemented part of the pre-analysis module of input sequences. GRL helped set up the BatchPrimer3 server. JD and ODA participated the design and coordination, and helped to draft the manuscript. All authors read and approved the final manuscript.

## Supplementary Material

Additional file 1 BatchPrimer3 application with source code (batchprimer3.tar.gz). This is a tarred and gzipped file, in which there are two directories, "batchprimer3_cgi-bin" and "batchprimer3_htdocs", and a README.txt file for installation instructions. Click here for file
