# Supplementary material for: BatchPrimer3: A high throughput web application for PCR and sequencing primer design
Source: BMC Bioinformatics. 2008 May 29;9:253. doi: 10.1186/1471-2105-9-253 (PMC2438325; doi:10.1186/1471-2105-9-253)
Supplement: Additional file 1 — BatchPrimer3 application with source code (batchprimer3.tar.gz). This is a tarred and gzipped file, in which there are two directories, "batchprimer3_cgi-bin" and "batchprimer3_htdocs", and a README.txt file for installation instructions. [file 1471-2105-9-253-S1.gz › batchprimer3/batchprimer3_htdocs/menu.html]

BatchPrimer3


|  |  |
| --- | --- |
| |  | | --- | | **BatchPrimer3  v1.0** | |
| **Overview** |
| **Primer Design Server** |
| **Primer Resources** |

**User Statistics  
since Oct. 21, 2007**

|  |
| --- |
| Last modified on  October 18, 2007. Comments and Questions:  Dr. Frank Mingan You  frankyou@pw.usda.gov |


|  |
| --- |
| Department of Plant Sciences  University of California, Davis   One Shields Avenue • Davis, CA 95616  Genomics & Gene Discovery  USDA-ARS, WRRC  800 Buchanan Street • Albany, CA 94710 |
