# Supplementary material for: BatchPrimer3: A high throughput web application for PCR and sequencing primer design
Source: BMC Bioinformatics. 2008 May 29;9:253. doi: 10.1186/1471-2105-9-253 (PMC2438325; doi:10.1186/1471-2105-9-253)
Supplement: Additional file 1 — BatchPrimer3 application with source code (batchprimer3.tar.gz). This is a tarred and gzipped file, in which there are two directories, "batchprimer3_cgi-bin" and "batchprimer3_htdocs", and a README.txt file for installation instructions. [file 1471-2105-9-253-S1.gz › batchprimer3/batchprimer3_htdocs/overview.html]

Overview of BatchPrimer3


|  |  |  |  |
| --- | --- | --- | --- |
| |  | | --- | |  | | BatchPrimer3 v1.0 | | A high throughput web application for PCR and sequencing primer design | |
|  |
| BatchPrimer3 is a comprehensive web primer design program using Primer3 core program as a major primer design engine to design different types of PCR primers and sequencing primers in a high-through manner. BatchPrimer3 allows users to design several types of primer designs including generic primers, hybridization oligos, SSR primers together with SSR detection, and SNP genotyping primers (including single-base extension primers, allele-specific primers, and tetra-primers for tetra-primer ARMS PCR), as well as DNA sequencing primers. A batch input of large number of sequences and a tab-delimited result output greatly facilitates rapid primer design and ordering process. Primer design server Click here to go to the primer design server page Primer design methodsIn BatchPrimer3 1.0, several types of primer design are implemented:  - ***Generic primer design***: The same features as Primer3 Web (Rozen and Skaletsky, 2000)   are provided to design pairs of prim-ers based on any DNA sequences.- ***SNP flanking primer design***: Design pairs of primers that flank the SNP site.- ***Single base extension (SBE) primer design***: SBE is a technique for detecting       known SNP site. Design a primer which anneals immediately adjacent to the SNP       is extended by one base using a fluorescently labeled ddNTP. Design primers that       anneal immedi-ately next to the SNP site. Two best SNP primers, one for each       orientation (forward and reverse), are designed for user selection according to scores.- ***Allele-specific primer design***: Designs primers that will specifi-cally amplify one of         the alleles. Two primers that flank the alleles are picked. For each orientation         (forward and reverse) two best allele primers can be selected from up to four allele         primers (two for each orientation). Primer3 core program is used to pick the allele         flanking primers and the similar algorithm for SNP primer design is used to         choose the best allele primers.- ***SNP flanking primer and single base extension primer design***: Design pairs of           primers that flank the SNP site and a SNP primer that neighbor or include the SNP nucleotide.- ***Allele-specific primer design***: Designs primers that will specifically             amplify one of the alleles. Two primers that flank the alleles are picked. For each             orientation (forward and reverse) two best allele primers can be selected from up to             four allele primers (two for each orientation). Primer3 core program is used to pick             the allele flanking primers and the similar algorithm for SNP primer design is used to             choose the best allele primers.- ***Tetra-primer ARMS PCR primers***:               Ye et al. (2001) proposed a simple, effective and economical SNP genotyping method based on AS primers called tetra-primer ARMS-PCR.               This procedure adopts principles of the tetra-primer PCR method and the amplification refractory mutation system (ARMS). Four primers are required               to amplify a larger fragment from template DNA containing the SNP and two smaller fragments representing each of the two AS products.               Primers are designed in such a way that the amplicons of two alleles differ in sizes and can be resolved by agarose gel electrophoresis.               To enhance the specificity of the reaction, in addition to the first mismatch at the 3' end of AS primers, an extra mismatch is also deliberately introduced               at the third position from the 3' end of each of the two inner AS primers. From the primer design perspective, two sets of tetra-primers for any SNP               can be designed theoretically according to AS primer orientation. BatchPrimer3 v1.0 implemented a batch module to easily design two sets of tetra-primers for a SNP.- ***SSR screening and primer design***: SSR or microsatellite is a simple sequence repeat, which                 is a useful genetic marker. SSR primers are picked from the SSR-flanking regions.                 SSR motif screening varies in criteria of SSR definition, i.e., motif length and                 number of motif repeats or SSR length. Typically dinucleotide motifs to hexanucleotide                 motifs are detected with at least 12 nucleotides in length of SSRs. Since the                 different criteria of SSR screening will result in large difference in screening results,                 BatchPrimer3 provides flexible options to allow users to set the screening criteria.                 A regular expression was used to design an al-gorithm to detect the repeat motifs and                 number of repeats. The detected SSRs are masked as targets. Then Primer3 program picks                 the best pairs of primers flanking the targets.- ***Hybridization oligo design***: Design hybridization oligo primers,                   which is the same as in Primer3 Web.- ***Sequencing primers***:                     DNA samples can be sequenced in either the forward or reverse direction, or both. Candidates of a forward sequencing primer are                     scanned starting from the 5' end of target sequence until a primer meeting the user�s parameter settings with a high quality score (greater than or equal to 60) is found.                     The same procedure is applied to selection of reverse sequencing primer except the starting point of primer scanning is the 3' end of target sequence.  Sequence InputTwo ways are available to input sequences:  1. Sequences can be copied and then pasted to a sequence text box. This approach has a    maximum 256 kb size limit.- A FASTA file can be uploaded to the server and the sequence size limitation only depends on      Internet speed and server machine memory.  A FASTA format is acceptable in the BatchPrimer3 program. See example.  - For SNP flanking primers or allele-specific primer design, the SNPs or alleles in the   sequence must be masked using IUB/IUPAC nucleic acid code (G/C→S, A/T→W, G/A→R, T/C→Y, G/T→K,   A/C→M), and the sequence file follows the NCBI dbSNP FASTA format. See example.- For generic primer and oligo primer desgin, the “[]” pair can be used to specify targets,     the “{}” pair to specify included region, and the “<>” pair to specify excluded region.     The program can recognize them and automatically determine the proper parameters to pick     primers to flank targets, or exclude the specified regions or only include the specified     region for primer design. See example.  Sequence pre-analysisA utility tool for pre-analysis of input sequences is provided in BatchPrimer3 to help users to understand the basic statistics of input sequences, such as sequence length, GC content and their distributions in a whole set of input sequences. The information provides hints to adjust the parameter ranges of product size and GC content. See example.Result outputThe BatchPrimer3 program produces four parts of outputs:  1. a main HTML page containing the primer design summary of all input sequences;- an HTML table page listing all designed primers and primer properties (see example);- a tab-delimited text file with the same contents in the HTML table page,- and a detailed primer view page for each sequence with successfully designed primers (see example).          A simple click on the links on the main HTML page or HTML table page will display the primer view.          The primer list can be directly saved as a text file or an Excel file for further editing or primer ordering. |
