# Supplementary material for: BatchPrimer3: A high throughput web application for PCR and sequencing primer design
Source: BMC Bioinformatics. 2008 May 29;9:253. doi: 10.1186/1471-2105-9-253 (PMC2438325; doi:10.1186/1471-2105-9-253)
Supplement: Additional file 1 — BatchPrimer3 application with source code (batchprimer3.tar.gz). This is a tarred and gzipped file, in which there are two directories, "batchprimer3_cgi-bin" and "batchprimer3_htdocs", and a README.txt file for installation instructions. [file 1471-2105-9-253-S1.gz › batchprimer3/batchprimer3_htdocs/sequencing_primer_design_notes.pdf]

Notes of Sequencing Primer Design

Frank You, 6/29/2007

Objectives

Design a set of sequencing primers from EST sequences that as markers are associated with some BAC clones and contigs of D genome physical maps. These primers should be picked from the regions close to the stop codon or 3'UTR regions of genes.

Results:

1. From the unique 742 5' EST sequences that are associated with clones and contigs of D genome physical maps, a total of 545 3' EST sequences corresponding to 5' EST in a same probe are obtained.
2. Blastx search against NCBI non-redundant protein database (nr) found that 236 3' EST sequences hit proteins, and that only 192 out of 236 3' EST sequences aligned to the end (i.e., stop codon or 3' UTR region) of protein sequences (genes).
3. Using BatchPrimer3 web program (http://wheat.pw.usda.gov/demos/BatchPrimer3/), 154 sequencing primers from 192 3' EST sequences are designed. The primers are listed in the attached excel file "final_primer_list.xls". The file contains 14 columns and their meanings are list in Table 1. The primers were picked according to the specified parameters in primer length, Tm, GC% and others. If the parameters are changed, you may get a different set of primers. The more stringent primers may result in fewer primers picked. All the primers are listed in the Excel file.

Table 1: Columns of primer list file

Column Name	Meaning
5' EST	5'EST accession.
3' EST	3' EST accession corresponding to the 5' EST sequences in a same probe.
Bin	Wheat bin map location of 5' EST mapped to.
Primer Orientation	Primer orientation in primer design. All primers are designed in a REVERSE direction because the primers are close to STOP codon of a gene.
Start Pos	Primer start position. For reverse primers, the start position is counted from the right side of sequences. A negative value is assigned counting from the end of the sequence. This tells you how far a primer is picked from 3' UTR.
Primer Length	Primer sequence length.

Tm	Primer melting temperature.
GC%	Primer GC content
Q Score	Quality score of primer. This score is calculated to rank the candidate primers when primers are picked from sequences. Primer Q score must be greater or equal to 70.
Primer Sequence	Primer sequence.
Clones	Clone list that the 5' EST is associated with.
Contigs	Contig list that the 5' EST is associated with.
Protein Accession	Protein accession that 3' EST is aligned to the end of protein sequence.
Annotation	Annotation information of the protein: gene name and species etc.

Methods:

EST markers:

A list of EST markers associated with some clones and contigs in wheat D genome physical mapping project were extracted. After some edition by Ming-cheng Luo, a new list of markers with 1139 records and 742 unique ESTs were obtained. Most of EST markers are from 5' EST sequences, and only 10 of them are 3' EST sequences (BQxxxxxx). I forgot to put these 10 3'EST sequences in analysis. More sequencing primers might be designed from this sequences if necessary in future.

Used scripts:

unique_est.pl: create a unique EST accession list

unique_markers_ctgs.pl: create a file with unique EST accession, marker list, and contig lists.

3' EST sequences:

To find the UTR region, we used 3' EST sequences to identify 3' UTR and design primers. A total of 14582 3' EST sequences are extracted from wEST databases using the following sql script:

```
select distinct
  5prime.acc as 5prime,
  3prime.acc as 3prime,
  map_bin.name as bin,
  3prime.seq as 3prime_seq
from map_locus_rev
inner join est as 5prime on map_locus_rev.est_id = 5prime.id
inner join est_seqdir on 5prime.id = est_seqdir.fiveprime_est_id
inner join est as 3prime on est_seqdir.threeprime_est_id = 3prime.id
```

```
inner join map_bin on map_locus_rev.map_bin_id = map_bin.id
inner join est_lib on 5prime.est_lib_id = est_lib.id
Where est_lib.genus = 'Triticum'
order by map_bin.name
```

A total of 545 3' EST sequences corresponding to the 5' EST sequences in 5' EST marker table are extracted from the above sequences and used to next step of analysis.

Used scripts:

extract_seq.pl: extract unique 3' EST sequences.

Identification of stop codon or 3' UTR region:

1. The NCBI non-redundant protein database (nr) was downloaded from the NCBI FTP site at ftp://ftp.ncbi.nih.gov/blast/db.
2. A blastx search against nr database was performed. The blast report file was parsed to determine an EST is aligned to the end of a protein. If an EST sequences is aligned to the end of a protein sequence, we believe the EST sequence has a STOP codon and 3' UTR region in the EST sequence. This can be verified and conformed by several hits to different proteins. The information about STOP codon location in the EST sequences is extracted.

Used scripts:

Blast2table_plus.pl: convert the blast report into table format. Extra two columns, length of query sequence (EST sequences in this case) and length of subject sequence (protein in this case) are added to the table. These two columns will be used to identify whether or not EST sequences match to the end of protein sequences.

Blastx_table_process.pl: process the converted blast table data and create a file with stop codon coordinates in EST sequences.

Identification of splice sites in EST sequences:

To avoid the primers picked from EST sequences that span across splice sites, we must identify whether or not there are splice site existing in EST sequences. This information can be directly obtained from the annotation data of a hit protein.

1. Create a protein accession list file.
2. Use NCBI batch Entrez tool (http://www.ncbi.nlm.nih.gov/entrez/batchentrez.cgi?db=Nucleotide) to search the hit proteins and save results in GenPept format to a file.
3. Parse the GenPept data of each protein and extract CDS information to a file

Example of CDS in a hit protein:

```
LOCUS       CAB45912                 329 aa            linear   PLN 14-NOV-2006
DEFINITION  putative protein [Arabidopsis thaliana].
ACCESSION   CAB45912
VERSION     CAB45912.1  GI:5262764
DBSOURCE    embl accession AL080283.1
KEYWORDS    .
```

```
SOURCE      Arabidopsis thaliana (thale cress)
  ORGANISM  Arabidopsis thaliana
            Eukaryota; Viridiplantae; Streptophyta; Embryophyta; Tracheophyta;
            Spermatophyta; Magnoliophyta; eudicotyledons; core eudicotyledons;
            rosids; eurosids II; Brassicales; Brassicaceae; Arabidopsis.
REFERENCE   1
  AUTHORS   Bevan,M., Murphy,G., Ridley,P., Hudson,S., Bancroft,I., Mewes,H.W.,
            Mayer,K.F.X., Lemcke,K. and Schueller,C.
  JOURNAL   Unpublished
REFERENCE   2  (residues 1 to 329)
  AUTHORS   EU Arabidopsis sequencing,project.
  TITLE     Direct Submission
  JOURNAL   Submitted (24-JUN-1999) MIPS, at the Max-Planck-Institut fuer
            Biochemie, Am Klopferspitz 18a, D-82152 Martinsried, FRG, E-mail:
            schuelle@mips.biochem.mpg.de,mayer@mips.biochem.mpg.de Project
            Coordinator: Mike Bevan, Molecular Genetics Department, Cambridge
            Laboratory, John Innes Centre, Colney Lane, NR4 7UJ Norwich, UK,
            E-mail: michael.bevan@bbsrc.ac.uk
COMMENT     Information on performance of analysis and a more detailed
            annotation of this entry and other sequences of chromosomes 3, 4
            and 5 can be viewed at: http://www.mips.biochem.mpg.de/proj/thal/.

FEATURES             Location/Qualifiers
     source          1..329
                     /organism="Arabidopsis thaliana"
                     /variety="Columbia"
                     /db_xref="taxon:3702"
                     /chromosome="4"
                     /ecotype="Columbia"
     Protein         1..329
                     /product="putative protein"
     Region          93..327
                     /region_name="WcaG"
                     /note="Nucleoside-diphosphate-sugar epimerases [Cell
                     envelope biogenesis, outer membrane / Carbohydrate
                     transport and metabolism]; COG0451"
                     /db_xref="CDD:30800"
     CDS             1..329
                     /gene="F3L17.100"
                     /coded_by="join(AL080283.1:60160..60415,
                     AL080283.1:60515..60627,AL080283.1:60704..60757,
                     AL080283.1:60834..60918,AL080283.1:61041..61146,
                     AL080283.1:61234..61335,AL080283.1:61423..61520,
                     AL080283.1:61602..61711,AL080283.1:61878..61943)"
                     /note="similarity to hypothetical protein - Arabidopsis
                     thaliana, PIR2:T04873
                     contains EST gb:AA721947, T41838, Z30798"
                     /db_xref="GOA:Q9SV17"
                     /db_xref="InterPro:IPR001509"
                     /db_xref="UniProtKB/TrEMBL:Q9SV17"
ORIGIN
        1 matttnlsfa ppsysrfaat ksqirnplft splplpssff lvrneaslss sitpvqafte
       61 eavdtsdlas sssklvlvvg gtggvgqlvv asllkrnirs rlllrdldka tklfgkqdey
      121 slqvvkgdtr naedldpsmf egvthvictt gttafpskrw neentpekvd wegvknlisa
      181 lpssvkrvvl vssvgvtksn elpwsimnlf gvlkykkmge dflrdsglpf tiirfrtkep
      241 grltdgpyts ydlntllkat agerravvig qgdnlvgevs rlvvaeaciq aldieftqgk
      301 ayeinsvkgd gpgsdpqqwr elfkaaesk
//
```

The blue part will be parsed to get splice site information.

Used scripts:

Extract_cds.pl: parse the GenPept file and extract CDS information (splice site coordinate).

Extract final 3' sequences with STOP codon and splice sites for primer design:

According to 3'UTR and splice site information, the final 3' EST sequences were extracted from 545 3' EST sequences. Only pieces of sequences which match protein and

are aligned to the end of protein are extracted. If there is any splice site in sequences, ten N's are inserted into the sequence. This will avoid picking primers across splice sites. Here number of N's inserted is not important for sequencing primers because we don't need to predict product size in pair of primer design.

Because 6 – reading frame translation probably cause some difference in splice site coordinates from protein sequence to DNA sequence, the 10 nucleotides at both sides of a splice site from EST sequences were chopped to make sure that the extracted pieces sequences do not contain splice site.

Here is an example of the final sequence for primer design:

Before processing:

```
>BQ162199
TCAGCTGAGGCAAGCTAAAGACGAAGTAGATAAGCCAGGGCTTCAAGTAA
TGCTTCAAAAGGTGTTGCAACTATATGCTTCCAACTTTCTCCGAAAGCGC
            ^splice site1
AGTTACGCTTATAAAGGGGGAGAGGTTGTAGTGCCTGAAAAGTTTCTTGA
                                ^splice site 2 at 128
ATCGATAATAGAGGCTCCCGAAAATGACTGGAATAGGCTGTTGCTTGATG   CDS
GACTTACAGTTGGAAAGGGAGATGTTTCACCTGAAGAATTTTACGCTGTT
                                     ^splice site 3 at 233
ACCAAGAAGAGAATTGAGAGAATCTTGATTCGCACGGAAGGAGGTTCTTA
TCAGCAACGGGTACTTGTCGAATATATAAAAGAGATACAAGCTAGAGCAG
              ^splice site 4 at 314
AGGAAATAGTGAACCGGCTTCAAGGCCCAGCTGTGTAACGTTTATGGTAC
ATTTGTAGTTACTGAAAAAGGCTTTCGCCCTGCTTTATATATAAAGCACA   3'UTR region
ATCCACAACAACACGGTACAAACGCACGCCACCAAAAAAAAAAAAAAAAA
AAA
```

Note: The red color is coding region. The pieces with blue color will be chopped.

After processing:

```
>BQ162199
AGCTGAGGCAAGCTAAAGACGAAGTAGATAAGCCAGGGCTTCAAGTAATG
NNNNNNNNNNATATGCTTCCAACTTTCTCCGAAAGCGCAGTTACGCTTAT
AAAGGGNNNNNNNNNNAAAGTTTCTTGAATCGATAATAGAGGCTCCCGAA
AATGACTGGAATAGGCTGTTGCTTGATGGACTTACAGTTGGAAAGGGAGA
TNNNNNNNNNNCGCTGTTACCAAGAAGAGAATTGAGAGAATCTTGATTCG
CACGGAAGGAGGTTCTTATCAGNNNNNNNNNNTATAAAAGAGATACAAGC
                      Intron of some size
TAGAGCAGAGGAAATAGTGAACCGGCTTCAAGGCCCAGCTGTG
                        ←------------------------------3' UTR
```

Used scripts:

Edit_splices.pl: edit the splice information and convert the coordinates of splice site in protein sequences into the coordinates in EST sequences.

Extract_final_seqs.pl: based on the STOP codon and splice site information extract the final sequences to a FASTA file for primer design.

Sequencing primer design:

1. Primer design program: BatchPrimer3 web program (http://wheat.pw.usda.gov/demos/BatchPrimer3/) was used to design sequencing primers. A new program module for sequencing primer was added to this web program package. A manuscript about this web primer design program is in preparation. The primers are picked in the region closed to end of sequence or STOP codon (Figure 1).

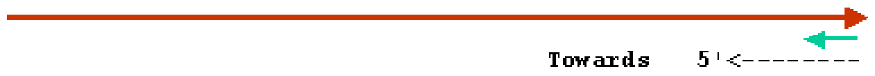

Figure 1. Reverse sequencing primer design in Pimer3Batch

2. Parameters used in primer design (Table 2):

Table 2: Parameters used in sequencing primer design

Parameter	Value
Orientation	Reverse, toward 5'
Primer length	20-28 nt, optimum 24 nt
Tm	57°C-63°C, optimum 60 °C
GC%	40%-60%
Primer complementarity	<= 8
3' complementarity	<= 3
Maximum N's	0, no ambiguity bases are allowed
Salt concentration	50 nM
DNA concentration	50 nM

Used program and scripts:

BatchPrimer3: design sequencing primers and save the results as a tab-limited file.

Merge_to_final_primer_list.pl: merge primer list with other information (5'EST name, bin location, protein hit and annotation, associated clones and contigs etc.).
