# Supplementary material for: BatchPrimer3: A high throughput web application for PCR and sequencing primer design
Source: BMC Bioinformatics. 2008 May 29;9:253. doi: 10.1186/1471-2105-9-253 (PMC2438325; doi:10.1186/1471-2105-9-253)
Supplement: Additional file 1 — BatchPrimer3 application with source code (batchprimer3.tar.gz). This is a tarred and gzipped file, in which there are two directories, "batchprimer3_cgi-bin" and "batchprimer3_htdocs", and a README.txt file for installation instructions. [file 1471-2105-9-253-S1.gz › batchprimer3/batchprimer3_htdocs/disclaimer.html]

Copyright notice and disclaimer of Primer3


|  |
| --- |
| Copywright Notice and Disclamer of Primer3Copyright (c) 1996,1997,1998 Whitehead Institute for Biomedical Research. All rights reserved. Redistribution and use in source and binary forms, with or without modification, are permitted provided that the following conditions are met:  1. Redistributions must reproduce the above copyright notice, this    list of conditions and the following disclaimer in the documentation    and/or other materials provided with the distribution. Redistributions of    source code must also reproduce this information in the source code itself.- If the program is modified, redistributions must include a notice      (in the same places as above) indicating that the redistributed program is      not identical to the version distributed by Whitehead Institute.- All advertising materials mentioning features or use of this        software must display the following acknowledgment: *This product includes software developed by the        Whitehead Institute for Biomedical Research.* - The name of the Whitehead Institute may not be used to endorse or          promote products derived from this software without specific prior written          permission.  We also request that use of this software be cited in publications as *Steve Rozen, Helen J. Skaletsky (1998) Primer3. Code available at http://primer3.sourceforge.net/.* THIS SOFTWARE IS PROVIDED BY THE WHITEHEAD INSTITUTE ``AS IS'' AND ANY EXPRESS OR IMPLIED WARRANTIES, INCLUDING, BUT NOT LIMITED TO, THE IMPLIED WARRANTIES OF MERCHANTABILITY AND FITNESS FOR A PARTICULAR PURPOSE ARE DISCLAIMED. IN NO EVENT SHALL THE WHITEHEAD INSTITUTE BE LIABLE FOR ANY DIRECT, INDIRECT, INCIDENTAL, SPECIAL, EXEMPLARY, OR CONSEQUENTIAL DAMAGES (INCLUDING, BUT NOT LIMITED TO, PROCUREMENT OF SUBSTITUTE GOODS OR SERVICES; LOSS OF USE, DATA, OR PROFITS; OR BUSINESS INTERRUPTION) HOWEVER CAUSED AND ON ANY THEORY OF LIABILITY, WHETHER IN CONTRACT, STRICT LIABILITY, OR TORT (INCLUDING NEGLIGENCE OR OTHERWISE) ARISING IN ANY WAY OUT OF THE USE OF THIS SOFTWARE, EVEN IF ADVISED OF THE POSSIBILITY OF SUCH DAMAGE. |
