# Supplementary material for: BatchPrimer3: A high throughput web application for PCR and sequencing primer design
Source: BMC Bioinformatics. 2008 May 29;9:253. doi: 10.1186/1471-2105-9-253 (PMC2438325; doi:10.1186/1471-2105-9-253)
Supplement: Additional file 1 — BatchPrimer3 application with source code (batchprimer3.tar.gz). This is a tarred and gzipped file, in which there are two directories, "batchprimer3_cgi-bin" and "batchprimer3_htdocs", and a README.txt file for installation instructions. [file 1471-2105-9-253-S1.gz › batchprimer3/batchprimer3_htdocs/primerdata.html]

Overview of BatchPrimer3


|  |  |  |
| --- | --- | --- |
| |  | | --- | |  | | Primer Resources | |
|  |
| Using the BatchPrimer3 program we have designed thousands of primers in several genomic research projects, including conserved intron-spanning primer pairs from EST sequences for wheat SNP discovery, SNP genotyping primers for wheat SNP mapping, standard primer pairs from *Brachypodium* bacteria artificial chromosome (BAC) end sequences for *Brachypodium* SNP discovery, sequencing primers from EST sequences for gene-specific sequencing, and SSR flanking primer pairs from *Brachypodium* EST and BAC end sequences for *Brachypodium* SSR genotyping. Most of these primers have been validated in experiments from several laboratories..   1. Wheat conserved intron-spanning primer sets.- Wheat SNP genotyping primer set from 1527 genome-specific loci      - Single base extension (SBE) primer sets- Allele-specific orimer sets- Tetra-primer sets for for tetra-primer ARMS PCR- Wheat sequencing primer design        - Notes of sequencing primer desing using EST sequences- Sequencing primer list- *Brachypodium* SSR detection and SSR-flanking primer sets. |
