# Supplementary material for: BatchPrimer3: A high throughput web application for PCR and sequencing primer design
Source: BMC Bioinformatics. 2008 May 29;9:253. doi: 10.1186/1471-2105-9-253 (PMC2438325; doi:10.1186/1471-2105-9-253)
Supplement: Additional file 1 — BatchPrimer3 application with source code (batchprimer3.tar.gz). This is a tarred and gzipped file, in which there are two directories, "batchprimer3_cgi-bin" and "batchprimer3_htdocs", and a README.txt file for installation instructions. [file 1471-2105-9-253-S1.gz › batchprimer3/batchprimer3_htdocs/batchprimer_results_help.html]

Help for report of high-throughput primer design


# Help for report of high-throughput primer design

Batch primer design program will output a list of primers for all user input sequences. If a user needs to view details of sequence with picked primers for a sequence, just click the Sequence ID, the full primer design results of the sequence will be available.

For each sequence, the results are reported using the following format. This is an example:

```
Sequence Index: 1
Sequence ID:    gnl|dbSNP|rs36234727|allelePos=301|totalLen=601|taxid=9606|snpclass=1|alleles='G/T'|mol=Genomic|build=126
                    Start  Len      Tm     GC%   any     3' Primer Sequence
1  LEFT PRIMER          4   18   59.75   61.11  4.00  0.00 GTGCAGGTGGAGGAGGAA
   RIGHT PRIMER       562   18   60.15   61.11  2.00  0.00 AGCCCTCTCCCCCTTTCT
   SEQUENCE SIZE: 601
   INCLUDED REGION SIZE: 601
   PRODUCT SIZE: 559, PAIR ANY COMPL: 7.00, PAIR 3' COMPL: 3.00


Allele-specific primers:Y (T/C)
                       Start  Len      Tm     GC%   Score Primer Sequence
   FORWARD 1             285   16   58.38   68.75   93.33 GGGGGAGAGGGCTCAT
   FORWARD 2             283   18   61.56   66.67   86.67 AAGGGGGAGAGGGCTCAC
   REVERSE 1             285   16   62.59   68.75   93.33 TGCACCCGCCTCCACT
```

In the rows of "LEFT PRIMER" and "RIGHT PRIMER", the columns are **Start** (Start position),
**len** (Oligo Length), **tm** (Melting Temperature), **gc%**, **any**
(Self Complementarity), **3'** (Self Complementarity), and **seq**
(Primer Sequence, 5'->3'), seperately. For SNP sinle base extension (SBE) primers or
allele-specific primers, score is also provided.

**start (Start Position)**: The position of the 5' base of the primer. For a Left Primer or Hyb Oligo this position is the position of the leftmost base. For a Right primer it is the position of the ***rightmost*** base. **len (Oligo Length)**: The length of the primer or oligo. **tm (Melting Temperature)**: The melting temperature of the primer or oligo. **gc%**: The percent of G or C bases in the primer or oligo. **any (Self Complementarity)**: The self-complementarity score of the oligo or primer (taken as a measure of its tendency to anneal to itself or form secondary structure). **3' (Self Complementarity)**: The 3' self-complementarity of the primer or oligo (taken as a measure of its tendency to form a primer-dimer with itself). **seq (Primer Sequence, 5'->3')**: The sequence of the selected primer or oligo, always 5'->3' so the right primer is on the opposite strand from the one supplied in the source input. (The right primer sequence is the sequence you would want synthesized in a primer order.) **scores**: The quality score of primer picked. The quality score is a function of primer length, number of single-base and three-base repeats, Tm, and number of an ambiguity code (N). If the Tm of a candidate is beyond the user-specified range, or if an ambiguity code exists next to SNP in the SNP primer se-quence, or the candidate contains repeats, the quality score is set to 0. The shorter primer candidate has higher score than longer one since oligo cost is directly associated with oligo size. The primer with the highest score is chosen. If the highest score is zero, no proper SNP primer is found.

For SNP sinle base extension (SBE) primers, one best primer is chosen in each direction.
If two primers are available in forward and reverse direction, a user can choose the best one
according to scores.

For allele-specific primers, one best primer for each allele of two is chosen in each direction.
Up to four best primers are provided for forward and reverse directions.
If four primers are available in forward and reverse direction, a user can choose the best two primers
for a pair of alleles according to scores.

---

BatchPrimer3 Web software is developed by Frank You
based on Primer3 core program and Primer3 Web from
Steve Rozen
steve@genome.wi.mit.edu
and 
Whitehead Institute/MIT Center for Genome Research.  
Last modified: October 17, 2007
