# Supplementary material for: BatchPrimer3: A high throughput web application for PCR and sequencing primer design
Source: BMC Bioinformatics. 2008 May 29;9:253. doi: 10.1186/1471-2105-9-253 (PMC2438325; doi:10.1186/1471-2105-9-253)
Supplement: Additional file 1 — BatchPrimer3 application with source code (batchprimer3.tar.gz). This is a tarred and gzipped file, in which there are two directories, "batchprimer3_cgi-bin" and "batchprimer3_htdocs", and a README.txt file for installation instructions. [file 1471-2105-9-253-S1.gz › batchprimer3/batchprimer3_htdocs/redirect.html]

BatchPrimer


## Redirection to BatchPrimer

---

**(probes.pw.usda.gov/batchprimer/)**
is the new web location.  


---
