# Supplementary material for: BatchPrimer3: A high throughput web application for PCR and sequencing primer design
Source: BMC Bioinformatics. 2008 May 29;9:253. doi: 10.1186/1471-2105-9-253 (PMC2438325; doi:10.1186/1471-2105-9-253)
Supplement: Additional file 1 — BatchPrimer3 application with source code (batchprimer3.tar.gz). This is a tarred and gzipped file, in which there are two directories, "batchprimer3_cgi-bin" and "batchprimer3_htdocs", and a README.txt file for installation instructions. [file 1471-2105-9-253-S1.gz › batchprimer3/batchprimer3_htdocs/batchprimer_ack.html]

Acknowledgements


# Acknowledgements

This BatchPrimer3 web site is developed by Frank You
based on the Primer3 and its web software Primer3Web (Rozen and Skaletskyi, 2000) and its enhanced version Primer3Plus (Untergasser et al., 2007). The online help document in Primer3Web is also adopted
and revised for BatchPrimer3.
The original Primer3Web software is provided by
*Steve Rozen*
steve\@genome.wi.mit.edu
and 
Whitehead Institute/MIT Center for Genome Research.
The development of Primer3 and the original Primer3Web site was funded by
Howard Hughes Medical Institute
and by the
National Institutes of Health,

National Human Genome Research Institute.
under grants R01-HG00257
(to David C. Page) and P50-HG00098 (to Eric S. Lander).

The original design of this primer-picking web site
by *Richard Resnick*
Primer3's design is heavily based on earlier
implementations of similar programs:
Primer 0.5 (*Steve Lincoln, Mark Daly, and Eric
S. Lander*) and Primer v2 (*Richard Resnick*).
*Lincoln Stein* championed the use of the Boulder-IO
format and the idea of
making the Primer3 engine a software component.
