# Supplementary material for: BatchPrimer3: A high throughput web application for PCR and sequencing primer design
Source: BMC Bioinformatics. 2008 May 29;9:253. doi: 10.1186/1471-2105-9-253 (PMC2438325; doi:10.1186/1471-2105-9-253)
Supplement: Additional file 1 — BatchPrimer3 application with source code (batchprimer3.tar.gz). This is a tarred and gzipped file, in which there are two directories, "batchprimer3_cgi-bin" and "batchprimer3_htdocs", and a README.txt file for installation instructions. [file 1471-2105-9-253-S1.gz › batchprimer3/batchprimer3_htdocs/batchprimer_help.html]

Input help of BatchPrimer3


# Input help of BatchPrimer3

## Cautions

Some of the most important issues in primer picking can be
addressed only before using Primer3. These are sequence quality
(including making sure the sequence is not vector and not
chimeric) and avoiding repetitive elements.

Techniques for avoiding problems include a thorough understanding
of possible vector contaminants and cloning artifacts coupled
with database searches using blast, fasta, or other similarity
searching program to screen for vector contaminants and possible
repeats. Repbase (J. Jurka, A.F.A. Smit, C. Pethiyagoda, and
others, 1995-1996,
ftp://ncbi.nlm.nih.gov/repository/repbase)
is an excellent source of repeat sequences and pointers to the
literature. Primer3 now allows you to screen candidate oligos
against a Mispriming Library (or a Mishyb Library in the case
of internal oligos).

Sequence quality can be controlled by manual trace viewing and
quality clipping or automatic quality clipping programs. Low-
quality bases should be changed to N's or can be made part of
Excluded Regions. The beginning of a sequencing read is often
problematic because of primer peaks, and the end of the read
often contains many low-quality or even meaningless called bases.
Therefore when picking primers from single-pass sequence it is
often best to use the Included Region parameter to ensure that
Primer3 chooses primers in the high quality region of the read.
In addition, Primer3 takes as input a
Sequence Quality
 list for
use with those base calling programs
such as Phred
(http://www.mbt.washington.edu/phrap\_documentation.html)
that output this information.

**Upload sequence file with FASTA format**: For batch primer design in a high-throughput manner, a text file with FASTA format can be loaded from the local machine of a user. If a user loads a file from the local machine, the program will process this file with highest priority even a user has copied and pasted sequences into the box. **Source Sequence**: The sequence from which to select primers or hybridization oligos. **Sequence Id**: An identifier that is reproduced in the output to enable you to identify the chosen primers. This is disabled in batch primer mode. You need to use FASTA format to provide sequence ids in the file or sequences. **E-mail address:**: An e-mail address can be optionally provided. The primer design report will be sent to the user if an email address is available. This is specifically for batch primer design of a large volume of sequences, which could take more than 5 minutes. In BatchPrimer3, an asynchronous mode with email alert was implemented. The job of primer design is executed in a separate thread. After the primer design job is finished, an email of result report will be sent to the user. **Targets**: If one or more Targets is specified then a legal primer pair must flank at least one of them. A Target might be a simple sequence repeat site (for example a CA repeat) or a single-base-pair polymorphism. The value should be a space-separated list of ``` start,length ``` pairs where *start* is the index of the first base of a Target, and *length* is its length. **Excluded Regions**: Primer oligos may not overlap any region specified in this tag. The associated value must be a space-separated list of ``` start,length ``` pairs where *start* is the index of the first base of the excluded region, and *length* is its length. This tag is useful for tasks such as excluding regions of low sequence quality or for excluding regions containing repetitive elements such as ALUs or LINEs. **Product Size**: Minimum, Optimum, and Maximum lengths (in bases) of the PCR product. Primer3 will not generate primers with products shorter than Min or longer than Max, and with default arguments Primer3 will attempt to pick primers producing products close to the Optimum length, **Number To Return**: The maximum number of primer pairs to return. Primer pairs returned are sorted by their "quality", in other words by the value of the objective function (where a lower number indicates a better primer pair). Caution: setting this parameter to a large value will increase running time. **Max 3' Stability**: The maximum stability for the five 3' bases of a left or right primer. Bigger numbers mean more stable 3' ends. The value is the maximum delta G for duplex disruption for the five 3' bases as calculated using the nearest neighbor parameters published in Breslauer, Frank, Bloeker and Marky, Proc. Natl. Acad. Sci. USA, vol 83, pp 3746-3750. Rychlik recommends a maximum value of 9 (Wojciech Rychlik, "Selection of Primers for Polymerase Chain Reaction" in BA White, Ed., "Methods in Molecular Biology, Vol. 15: PCR Protocols: Current Methods and Applications", 1993, pp 31-40, Humana Press, Totowa NJ). **Max Mispriming**: The maximum allowed weighted similarity with any sequence in Mispriming Library. Default is 12. **Pair Max Mispriming**: The maximum allowed sum of weighted similarities of a primer pair (one similarity for each primer) with any single sequence in Mispriming Library. Default is 24. **Primer Size**: Minimum, Optimum, and Maximum lengths (in bases) of a primer oligo. Primer3 will not pick primers shorter than Min or longer than Max, and with default arguments will attempt to pick primers close with size close to Opt. Min cannot be smaller than 1. Max cannot be larger than 36. (This limit is governed by maximum oligo size for which melting-temperature calculations are valid.) Min cannot be greater than Max. **Primer Tm**: Minimum, Optimum, and Maximum melting temperatures (Celsius) for a primer oligo. Primer3 will not pick oligos with temperatures smaller than Min or larger than Max, and with default conditions will try to pick primers with melting temperatures close to Opt. Primer3 uses the oligo melting temperature formula given in Rychlik, Spencer and Rhoads, Nucleic Acids Research, vol 18, num 12, pp 6409-6412 and Breslauer, Frank, Bloeker and Marky, Proc. Natl. Acad. Sci. USA, vol 83, pp 3746-3750. Please refer to the former paper for background discussion. **Maximum Tm Difference**: Maximum acceptable (unsigned) difference between the melting temperatures of the left and right primers. **Product Tm**: The minimum, optimum, and maximum melting temperature of the amplicon. Primer3 will not pick a product with melting temperature less than min or greater than max. If Opt is supplied and the Penalty Weights for Product Size are non-0 Primer3 will attempt to pick an amplicon with melting temperature close to Opt. Primer3 calculates product melting temperature using equation (iii) from Rychlik, Spencer and Rhoads, Nucleic Acids Research 18:21 pg. 6410. **Primer GC%** Minimum, Optimum, and Maximum percentage of Gs and Cs in any primer. **Max Complementarity**: The maximum allowable local alignment score when testing a single primer for (local) self-complementarity and the maximum allowable local alignment score when testing for complementarity between left and right primers. Local self-complementarity is taken to predict the tendency of primers to anneal to each other without necessarily causing self-priming in the PCR. The scoring system gives 1.00 for complementary bases, -0.25 for a match of any base (or N) with an N, -1.00 for a mismatch, and -2.00 for a gap. Only single-base-pair gaps are allowed. For example, the alignment ``` 5' ATCGNA 3' || | | 3' TA-CGT 5' ``` is allowed (and yields a score of 1.75), but the alignment ``` 5' ATCCGNA 3' || | | 3' TA--CGT 5' ``` is not considered. Scores are non-negative, and a score of 0.00 indicates that there is no reasonable local alignment between two oligos. **Max 3' Complementarity**: The maximum allowable 3'-anchored global alignment score when testing a single primer for self-complementarity, and the maximum allowable 3'-anchored global alignment score when testing for complementarity between left and right primers. The 3'-anchored global alignment score is taken to predict the likelihood of PCR-priming primer-dimers, for example ``` 5' ATGCCCTAGCTTCCGGATG 3' ||| ||||| 3' AAGTCCTACATTTAGCCTAGT 5' ``` or ``` 5` AGGCTATGGGCCTCGCGA 3' |||||| 3' AGCGCTCCGGGTATCGGA 5' ``` The scoring system is as for the Max Complementarity argument. In the examples above the scores are 7.00 and 6.00 respectively. Scores are non-negative, and a score of 0.00 indicates that there is no reasonable 3'-anchored global alignment between two oligos. In order to estimate 3'-anchored global alignments for candidate primers and primer pairs, Primer assumes that the sequence from which to choose primers is presented 5'->3'. It is nonsensical to provide a larger value for this parameter than for the Maximum (local) Complementarity parameter because the score of a local alignment will always be at least as great as the score of a global alignment. **Max Poly-X**: The maximum allowable length of a mononucleotide repeat, for example AAAAAA. **Included Region**: A sub-region of the given sequence in which to pick primers. For example, often the first dozen or so bases of a sequence are vector, and should be excluded from consideration. The value for this parameter has the form ``` start,length ``` where *start* is the index of the first base to consider, and *length* is the number of subsequent bases in the primer-picking region. **Start Codon Position**: This parameter should be considered EXPERIMENTAL at this point. Please check the output carefully; some erroneous inputs might cause an error in Primer3. Index of the first base of a start codon. This parameter allows Primer3 to select primer pairs to create in-frame amplicons e.g. to create a template for a fusion protein. Primer3 will attempt to select an in-frame left primer, ideally starting at or to the left of the start codon, or to the right if necessary. Negative values of this parameter are legal if the actual start codon is to the left of available sequence. If this parameter is non-negative Primer3 signals an error if the codon at the position specified by this parameter is not an ATG. A value less than or equal to -10^6 indicates that Primer3 should ignore this parameter. Primer3 selects the position of the right primer by scanning right from the left primer for a stop codon. Ideally the right primer will end at or after the stop codon. **Mispriming Library**: This selection indicates what mispriming library (if any) Primer3 should use to screen for interspersed repeats or for other sequence to avoid as a location for primers. **CG Clamp**: Require the specified number of consecutive Gs and Cs at the 3' end of both the left and right primer. (This parameter has no effect on the hybridization oligo if one is requested.) **Salt Concentration**: The millimolar concentration of salt (usually KCl) in the PCR. Primer3 uses this argument to calculate oligo melting temperatures. **Annealing Oligo Concentration**: The nanomolar concentration of annealing oligos in the PCR. Primer3 uses this argument to calculate oligo melting temperatures. The default (50nM) works well with the standard protocol used at the Whitehead/MIT Center for Genome Research--0.5 microliters of 20 micromolar concentration for each primer oligo in a 20 microliter reaction with 10 nanograms template, 0.025 units/microliter Taq polymerase in 0.1 mM each dNTP, 1.5mM MgCl2, 50mM KCl, 10mM Tris-HCL (pH 9.3) using 35 cycles with an annealing temperature of 56 degrees Celsius. This parameter corresponds to 'c' in Rychlik, Spencer and Rhoads' equation (ii) (Nucleic Acids Research, vol 18, num 12) where a suitable value (for a lower initial concentration of template) is "empirically determined". The value of this parameter is less than the actual concentration of oligos in the reaction because it is the concentration of annealing oligos, which in turn depends on the amount of template (including PCR product) in a given cycle. This concentration increases a great deal during a PCR; fortunately PCR seems quite robust for a variety of oligo melting temperatures. **Max Ns Accepted**: Maximum number of unknown bases (N) allowable in any primer. **Liberal Base**: This parameter provides a quick-and-dirty way to get Primer3 to accept IUB / IUPAC codes for ambiguous bases (i.e. by changing all unrecognized bases to N). If you wish to include an ambiguous base in an oligo, you must set Max Ns Accepted to a non-0 value. Perhaps '-' and '\* ' should be squeezed out rather than changed to 'N', but currently they simply get converted to N's. The authors invite user comments. **First Base Index**: The index of the first base in the input sequence. For input and output using 1-based indexing (such as that used in GenBank and to which many users are accustomed) set this parameter to 1. For input and output using 0-based indexing set this parameter to 0. (This parameter also affects the indexes in the contents of the files produced when the primer file flag is set.) In the WWW interface this parameter defaults to 1. **Inside Target Penalty**: Non-default values valid only for sequences with 0 or 1 target regions. If the primer is part of a pair that spans a target and overlaps the target, then multiply this value times the number of nucleotide positions by which the primer overlaps the (unique) target to get the 'position penalty'. The effect of this parameter is to allow Primer3 to include overlap with the target as a term in the objective function. **Outside Target Penalty**: Non-default values valid only for sequences with 0 or 1 target regions. If the primer is part of a pair that spans a target and does not overlap the target, then multiply this value times the number of nucleotide positions from the 3' end to the (unique) target to get the 'position penalty'. The effect of this parameter is to allow Primer3 to include nearness to the target as a term in the objective function. **Show Debuging Info**: Include the input to primer3\_core as part of the output.

## Sequence Quality

**Sequence Quality**: A list of space separated integers. There must be exactly one integer for each base in the Source Sequence if this argument is non-empty. High numbers indicate high confidence in the base call at that position and low numbers indicate low confidence in the base call at that position. **Min Sequence Quality**: The minimum sequence quality (as specified by Sequence Quality) allowed within a primer. **Min 3' Sequence Quality**: The minimum sequence quality (as specified by Sequence Quality) allowed within the 3' pentamer of a primer. **Sequence Quality Range Min**: The minimum legal sequence quality (used for interpreting Min Sequence Quality and Min 3' Sequence Quality). **Sequence Quality Range Max**: The maximum legal sequence quality (used for interpreting Min Sequence Quality and Min 3' Sequence Quality).

## Penalty Weights

This section describes "penalty weights", which allow
the user to modify the criteria that Primer3 uses
to select the "best" primers. There are two classes
of weights: for some parameters there is a 'Lt' (less
than) and a 'Gt' (greater than) weight. These
are the weights that Primer3 uses when the value
is less or greater than (respectively) the specified optimum.
The following parameters have both 'Lt' and 'Gt' weights:

- Product Size- Primer Size- Primer Tm- Product Tm- Primer GC%- Hyb Oligo Size- Hyb Oligo Tm- Hyb Oligo GC%

The Inside Target Penalty
and Outside Target Penalty
are similar, except that since they relate
to position they do not lend them selves to the
'Lt' and 'Gt' nomenclature.

For the remaining parameters the optimum is understood
and the actual value can only vary in one direction
from the optimum:

- Primer Self Complementarity- Primer 3' Self Complementarity- Primer #N's- Primer Mispriming Similarity- Primer Sequence Quality- Primer 3' Sequence Quality- Primer 3' Stability- Hyb Oligo Self Complementarity- Hyb Oligo 3' Self Complementarity- Hyb Oligo Mispriming Similarity- Hyb Oligo Sequence Quality- Hyb Oligo 3' Sequence Quality

The following are weights are treated specially:

Position Penalty Weight: Determines the overall weight of the position penalty in calculating the penalty for a primer. Primer Weight: Determines the weight of the 2 primer penalties in calculating the primer pair penalty. Hyb Oligo Weight: Determines the weight of the hyb oligo penalty in calculating the penalty of a primer pair plus hyb oligo.

The following govern the weight given to various
parameters of primer pairs (or primer pairs plus
hyb oligo).

- Tm difference- Primer-Primer Complementarity- Primer-Primer 3' Complementarity- Primer Pair Mispriming Similarity

## Hyb Oligos (Internal Oligos)

Parameters governing choice of internal
oligos are analogous to the parameters governing
choice of primer pairs.
The exception is Max 3' Complementarity
which is meaningless when applied
to internal oligos used for hybridization-based detection, since
primer-dimer will not occur. We recommend that Max 3' Complementarity
be set at least as high as Max Complementarity.
