# Supplementary figures and images for: BatchPrimer3: A high throughput web application for PCR and sequencing primer design
Source: BMC Bioinformatics. 2008 May 29;9:253. doi: 10.1186/1471-2105-9-253 (PMC2438325; doi:10.1186/1471-2105-9-253)

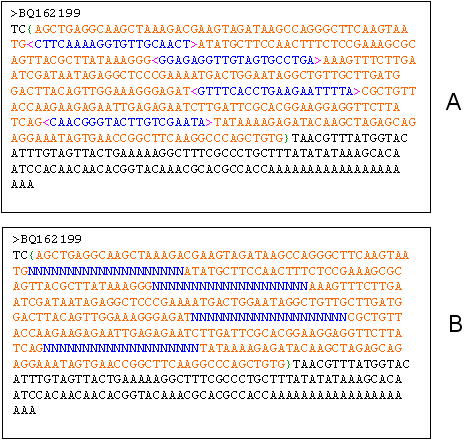

Supplement: Additional file 1 — BatchPrimer3 application with source code (batchprimer3.tar.gz). This is a tarred and gzipped file, in which there are two directories, "batchprimer3_cgi-bin" and "batchprimer3_htdocs", and a README.txt file for installation instructions. [file 1471-2105-9-253-S1.gz › batchprimer3/batchprimer3_htdocs/images/pre_processed.gif]

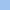

Supplement: Additional file 1 — BatchPrimer3 application with source code (batchprimer3.tar.gz). This is a tarred and gzipped file, in which there are two directories, "batchprimer3_cgi-bin" and "batchprimer3_htdocs", and a README.txt file for installation instructions. [file 1471-2105-9-253-S1.gz › batchprimer3/batchprimer3_htdocs/images/menu_bg.gif]

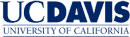

Supplement: Additional file 1 — BatchPrimer3 application with source code (batchprimer3.tar.gz). This is a tarred and gzipped file, in which there are two directories, "batchprimer3_cgi-bin" and "batchprimer3_htdocs", and a README.txt file for installation instructions. [file 1471-2105-9-253-S1.gz › batchprimer3/batchprimer3_htdocs/images/ucdavis_logo_2.gif]

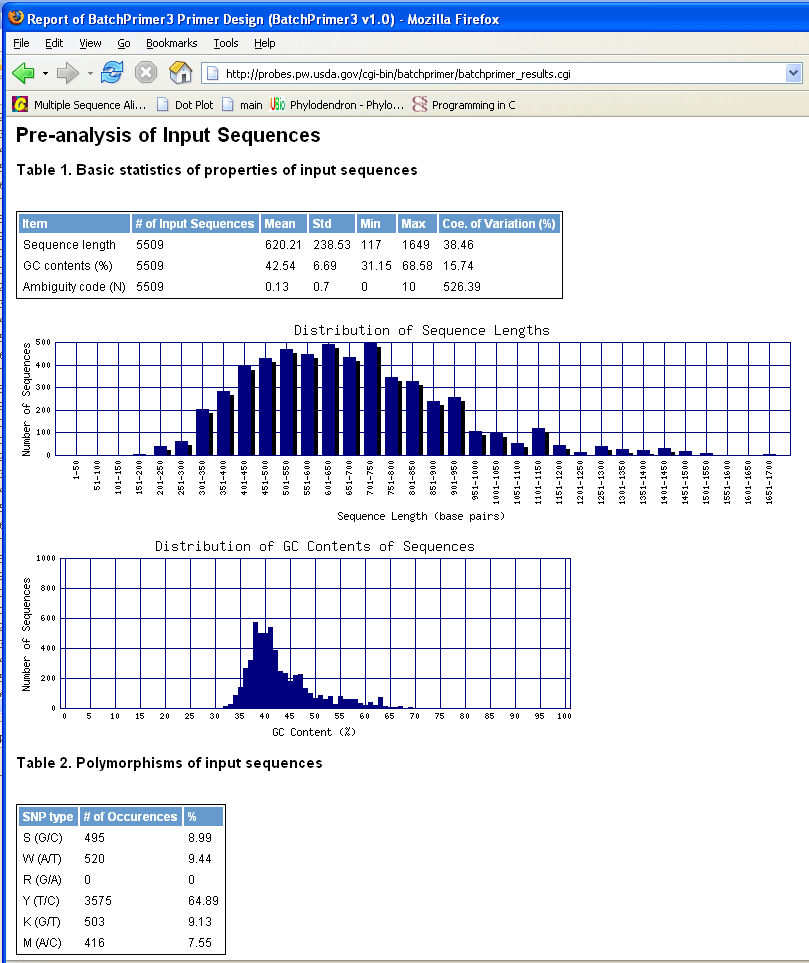

Supplement: Additional file 1 — BatchPrimer3 application with source code (batchprimer3.tar.gz). This is a tarred and gzipped file, in which there are two directories, "batchprimer3_cgi-bin" and "batchprimer3_htdocs", and a README.txt file for installation instructions. [file 1471-2105-9-253-S1.gz › batchprimer3/batchprimer3_htdocs/images/pre_analysis.gif]

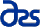

Supplement: Additional file 1 — BatchPrimer3 application with source code (batchprimer3.tar.gz). This is a tarred and gzipped file, in which there are two directories, "batchprimer3_cgi-bin" and "batchprimer3_htdocs", and a README.txt file for installation instructions. [file 1471-2105-9-253-S1.gz › batchprimer3/batchprimer3_htdocs/images/arslogo1-2.gif]

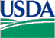

Supplement: Additional file 1 — BatchPrimer3 application with source code (batchprimer3.tar.gz). This is a tarred and gzipped file, in which there are two directories, "batchprimer3_cgi-bin" and "batchprimer3_htdocs", and a README.txt file for installation instructions. [file 1471-2105-9-253-S1.gz › batchprimer3/batchprimer3_htdocs/images/usdalogo1-1.gif]
